# Mathematical model of the Alzheimer’s disease biomarker cascade demonstrates statistical pitfall in identifying surrogates of cognitive reserve

**DOI:** 10.1016/j.isci.2024.111188

**Published:** 2024-10-18

**Authors:** Florian U. Fischer, Susanne Gerber, Oliver Tüscher, for the Alzheimer’s Disease Neuroimaging Initiative

**Affiliations:** 1 Department of Psychiatry and Psychotherapy, University Medical Center Mainz, Untere Zahlbacher Straße 8 D-55131 Mainz, Germany; 2 Institute of Human Genetics University Medical Center Mainz, Anselm-Franz-von-Bentzel-Weg 3 D-55128 Mainz, Germany; 3 Leibniz Institute for Resilience Research (LIR), Wallstraße 7 D-55122 Mainz, Germany

**Keywords:** Medicine, Pathology, Disease

## Abstract

Statistical interaction analyses with biomarkers of pathology on cognitive outcome have been put forward to investigate neurobiological surrogates of cognitive reserve in Alzheimer’s disease (AD). However, as these potential surrogates are likely affected by neurodegeneration during the course of AD, their joint alteration with biomarkers of pathology and cognitive outcome during disease progression may introduce misinterpretable interaction effects in cross-sectional studies. To demonstrate this, we conducted interaction analyses on synthetic data from a mathematical model of the AD biomarker cascade. When randomly sampling cross-sectionally, these gave interaction effects, which greatly reduced when controlling for the corresponding time point of each sampled data point. Cross-sectional studies investigating cognitive reserve using interaction analyses are advised to take into account the different time stages of the disease that individual data points represent.

## Highlights

• Mathematical model of the AD biomarker cascade without biomarker interactions
• Regression estimates of interactions of biomarkers on simulated data
• Sampling randomly or consistently determines magnitude of interaction estimate
• Interaction estimates may be misinterpretable under the biomarker cascade model

## Graphical abstract

**Figure:**
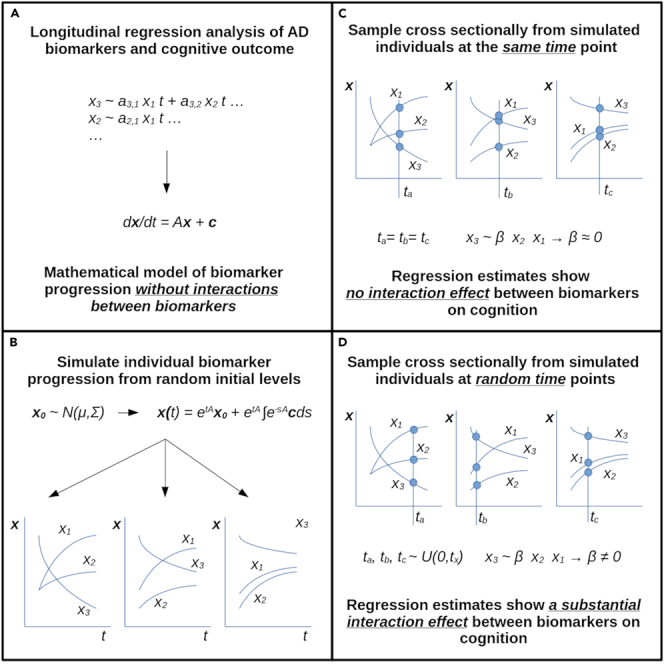

## Introduction

Individual trajectories of cognitive decline demonstrate considerable variance in the presence of comparable amounts of neuropathology associated with age-related neurodegenerative diseases, most commonly Alzheimer’s disease (AD). This has caused the exertion of considerable effort to conceptualize resilience to cognitive decline and its potential mechanisms as well as the identification and quantification of neurobiological bases thereof.^1^^,^^2^^,^^3^^,^^4^^,^^5^^,^^6^^,^^7^ According to the predominant concepts and nomenclature, the phenomenon of resilience has been trichotomized into brain maintenance, brain reserve, and cognitive reserve.^7^ Briefly, brain maintenance refers to the individually varied accumulation of age-related brain pathology. Brain reserve represents a quantifiable neurophysiological state of brain components, whose depletion over time by neuropathological processes to levels inducing cognitive impairment takes longer in individuals with higher starting quantities. Cognitive reserve on the other hand represents some form of functional adaption to the presence of neuropathology, modulating its impact on cognition. As the direct observation of cognitive reserve may be exceedingly difficult from an experimental design point of view, surrogates that refer to the neurophysiological prerequisites of cognitive reserve, such as e.g. white matter or regional gray matter volume etc., may be investigated. To this end, cognitive reserve has been operationalized statistically as a moderation effect of measures of pathology on cognition by reserve factors.^7^ However, as AD acts on many structures of the brain, such neurophysiological surrogates of cognitive reserve have a high chance of being affected as well, and thus arguably represent a convolute of effects of the disease process on the brain and information referring to the physiological basis of cognitive reserve. This raises the question of how the concepts of resilience are represented within the consensus model of AD. Extensive research has produced a biomarker cascade model for AD, wherein biomarkers are altered consecutively in a fixed order.^8^ The model includes cognitive outcome as its endpoint as well as biomarkers for molecular pathology, i.e. amyloid and tau, and biomarkers referring to neural injury to brain components such as hippocampal volume. Although findings of imaging studies from the last decade have considerably expanded our knowledge of the temporo-spatial associations of imaging biomarkers in the course of AD,^9^ this basic structure of the biomarker cascade model remains at the core of current conceptions of AD.^10^ This model incorporates cognitive reserve in the sense that the rate of change of cognitive outcome over time varies relative to biomarkers based on cognitive reserve. However, the possibility that some biomarkers may refer not only to neuronal injury due to AD but also to brain reserve or cognitive reserve, as, e.g., hippocampal volume,^6^ is not explicitly incorporated in the biomarker cascade model. If one argues that the cascade character of the model derives from time-lagged associations between the consecutively altered biomarkers where the rate of change of a later altered biomarker depends on the level of an earlier altered biomarker, accommodating initially varying individual biomarker configurations (representing brain maintenance or brain reserve) would simply require a corresponding shift of the biomarkers along the time axis. In addition, if the biomarkers contribute independently to the rate of cognitive decline, that rate would have to depend on the biomarkers accordingly. However, under the premise of this model, the identification of surrogates of cognitive reserve via statistical interaction effects^7^ may be problematic. Consider, for the sake of argument, a group of model subjects whose biomarker state trajectories are described by the model from the previous paragraph, where the initial configuration of biomarkers for the subjects varies randomly due to brain maintenance or brain reserve. We can make the following observations for the data points along these trajectories: (1) cognitive outcome will be lower at later time points, as the level of cognitive outcome depends on the amount of time it has been subject to the rate of change that is a function of biomarker levels. (2) All biomarkers themselves will also generally and thus jointly be more altered at later time points despite initially random distribution. If one were to randomly sample one data point from each trajectory to build a data sample, as is done in cross-sectional studies, and conduct statistical interaction analyses between a pair of biomarkers, one would find the following due to observations (1) and (2): data points, where only one of the main effect variables is altered will demonstrate less altered cognitive outcome, as they correspond to earlier time points from the trajectories. Data points, where both main effect variables are altered, will demonstrate more altered cognitive outcome as they correspond to later time points from the trajectories. A statistical analysis on such a sample is likely to report an interaction effect between biomarkers, which is an artifact produced by the underlying temporal dynamics of the biomarker trajectory, their initial variation, and the sampling procedure but could be mistakenly interpreted as an indicator of cognitive reserve.^7^ In order to test this hypothesis, we estimated a minimal mathematical model from empirical longitudinal data that formalizes the biomarker cascade model, accommodates individually differing initial biomarker configurations, and strictly additive, i.e. independent, effects of biomarkers on the rate of change of cognitive outcome. We then estimated regression models on the simulated data produced by the model, investigating possible faux interaction effects between biomarkers of molecular pathology, cerebral amyloid beta and corticospinal fluid (CSF) tau, and hippocampal volume, a biomarker that may partially refer to cognitive reserve surrogates.^6^ We propose a strategy to control for faux interaction effects in cross-sectional analyses of cognitive reserve. The findings of this study may inform the experimental design and inferences in studies investigating neurophysiological surrogates of brain reserve and cognitive reserve.

## Results

### Mathematical model parameters

For the model parameters *A* and $\vec{c}$ estimated by linear mixed-effects models, please refer to Table 1. Specifically, for the constant independent change of the biomarkers over time part of the model $\vec{c}$, an increase for amyloid positron emission tomography (PET) and CSF tau as well as a decrease for Alzheimer’s disease cognitive assessment (ADAS-cog) and hippocampal volume over time were estimated from empirical data. For the associations among biomarkers, higher amyloid-PET was associated with an increase of CSF tau and ADAS-cog as well as a decrease of hippocampal volume over time. Higher CSF tau was associated with increasing ADAS-cog over time. Finally, lower hippocampal volume was associated with increasing ADAS-cog over time.

**Table 1:** Estimated model parameters

$a_{i,j}$	ADAS-cog	HippoV	CSF tau	AV45	$c_{i}$
ADAS-cog	0	−0.23286	0.04579	0.11301	−0.08587
HippoV	0	0	0	−0.06553	−0.42319
CSF tau	0	0	0	0.01371	0.10595
AV45	0	0	0	0	0.19057

This table summarizes the model parameters $a_{i,j}\in A$ and $c_{i}\in \vec{c}$ estimated by linear mixed effects models. The elements of *A* quantify how much the biomarkers in the rows of the table change depending on the biomarkers in the columns. The elements of $\vec{c}$ in the last column quantify how much the biomarkers in the rows of the table change depending on time. ADAS-cog, cognitive Alzheimer’s disease assessment scale; HippoV, hippocampal volume; CSF tau, corticospinal fluid total tau; AV45, florbetapir amyloid-PET score.

### Agreement between empirical and simulated data

**Figure 1:**
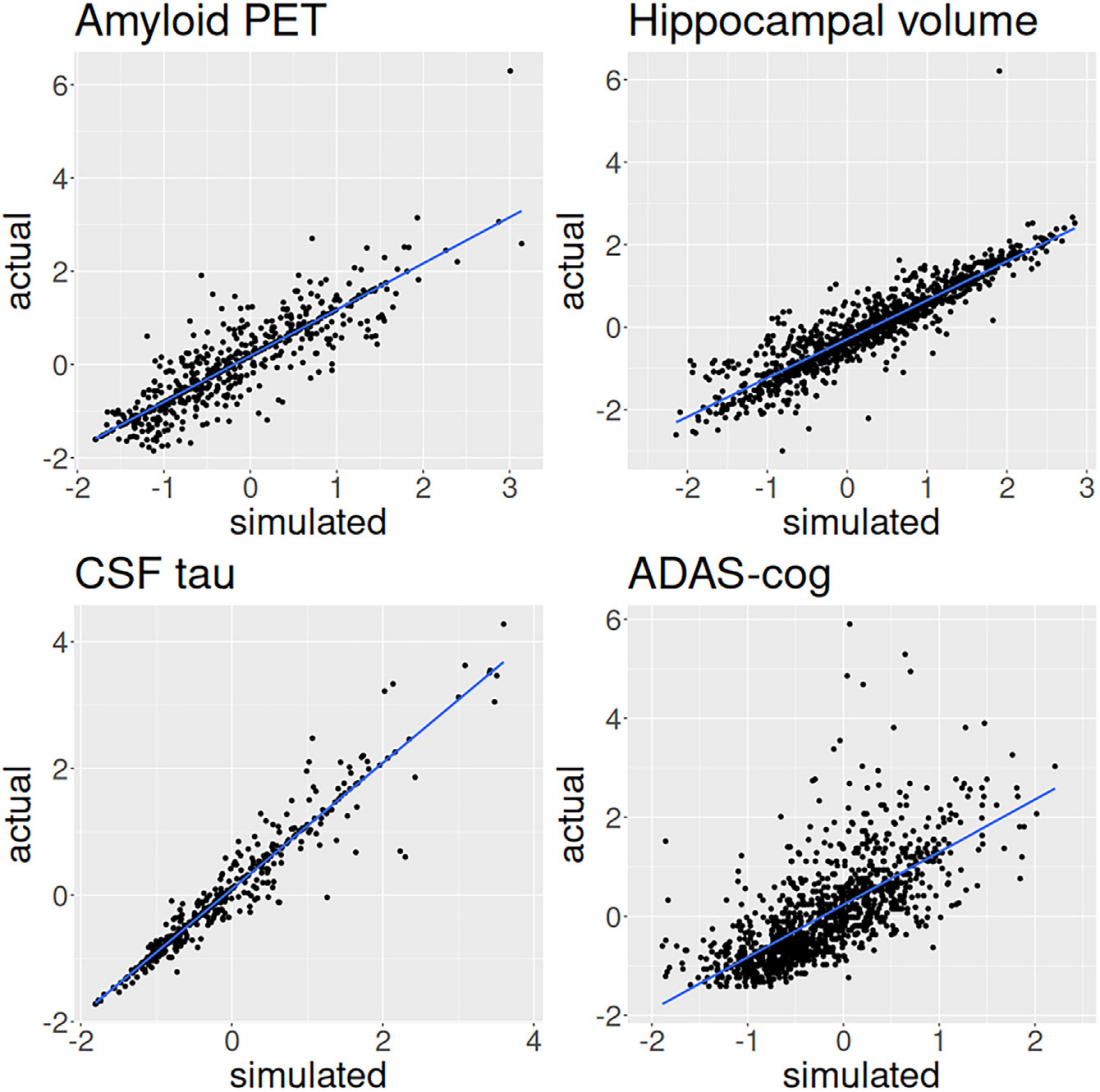
Actual versus simulated biomarker and cognitive outcome data points This plot shows the empirical data for all subjects at all available time points versus the corresponding simulated data points as predicted from the mathematical model with a line representing simple linear regression. CSF tau, corticospinal fluid total tau. ADAS-cog, cognitive Alzheimer’s disease assessment scale.

The estimated standardized coefficients of simple regression analyses between real and corresponding simulated data were 0.867, 0.977, 0.931, and 0.741 for amyloid-PET, CSF tau, hippocampal volume, and ADAS-cog, respectively. The corresponding adjusted $R^{2}$ values were 0.77, 0.92, 0.83, and 0.49. The simulated values thus showed substantial agreement with real data. See Figure 1 for plots of simple regression analyses.

### Emergence of faux interaction effect estimates due to data sampling

**Figure 2:**
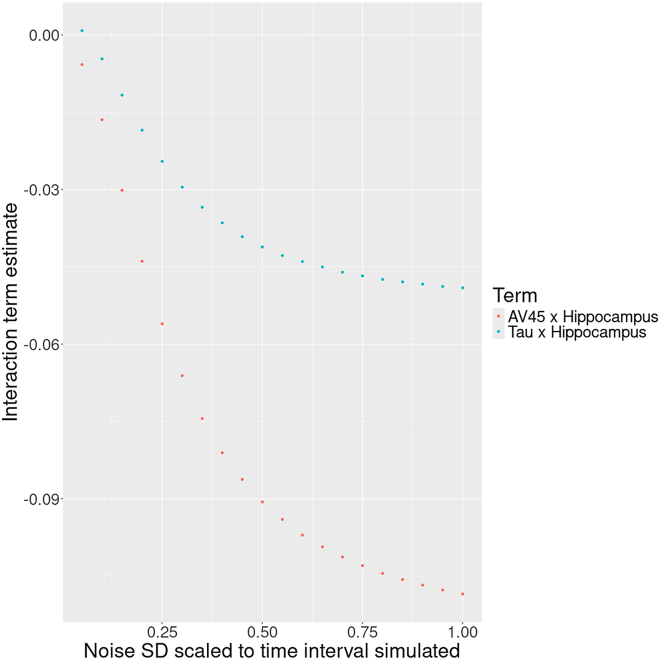
Interaction estimates on cross-sectional synthetic data when controlling with a noisy sampling time variable Shown on the x axis is the standard deviation, scaled to the data sampling time interval, of the Gaussian noise added to the data sampling time variable before partialing its variance out of regression variables. The y axis has averaged interaction term estimates between amyloid-PET (AV45) and CSF tau with hippocampal volume when predicting cognitive outcome in regression analyses on synthetic data simulated by the mathematical AD biomarker cascade model.

For the main analysis, data were simulated by the mathematical model that did not include interactions between biomarkers. Regression models yielded estimates of comparable magnitude for main effects (i.e., biomarkers) on cognitive outcome across the differently sampled simulated datasets. Specifically, for cross-sectional sampling that was consistent with respect to the time point of the data, the average standardized regression coefficients were 0.1891, 0.1371, and −0.7125 for amyloid-PET, CSF tau, and hippocampal volume, respectively. For cross-sectional sampling that was random with respect to the time point of the data, average standardized regression coefficients were 0.1790, 0.1228, and −0.7176 for amyloid-PET, CSF tau, and hippocampal volume, respectively. For cross-sectional sampling that was random with respect to the time point of the data and with the effect of time partialled out of variables, average standardized regression coefficients were 0.1962, 0.1344, and −0.7103 for amyloid-PET, CSF tau, and hippocampal volume, respectively. However, regression estimates of interaction effects differed between differently sampled simulated data samples. Specifically, for cross-sectional sampling that was consistent with respect to the time point of the data, average standardized regression coefficients were −0.0019 and 0.0038 for interactions of amyloid-PET and CSF tau with hippocampal volume, respectively. In contrast, for cross-sectional sampling that was random with respect to the time point of the data, average standardized regression coefficients were −0.1166 and −0.0526 for amyloid-PET with hippocampal volume and CSF tau with hippocampal volume, respectively. These interaction effects were such that at higher levels of hippocampal volume the detrimental effect of high amyloid-PET and CSF tau levels on cognitive outcome was attenuated. However, these interaction effects decreased when repeating the random sampling but partialing the effect of time out of variables prior to estimating regression models. The average standardized regression coefficients for this analysis were −0.0016 and 0.0028 for amyloid-PET with hippocampal volume and CSF tau with hippocampal volume, and thus comparable to those from the consistent sampling strategy. See Table 2 for an overview. Gaussian noise added to the time variable limited its effectiveness at suppressing the estimation of faux interaction effects: at 100% standard deviation in relation to the total time interval, interaction estimates were effectively on par with those when not partialing out time of sampling. With lowering standard deviation of noise, the interaction estimates decrease steadily and then more quickly from around 50%. Please see Figure 2 for a plot of this relationship.

**Table 2:** Main analysis regression estimates

Model term	Consistent	Random	Random f. residuals
AV45	0.1891 [0.1539,0.2102]	0.1790 [0.1767,0.1811]	0.1962 [0.1932,0.1993]
CSF tau	0.1371 [0.1002,0.1488]	0.1228 [0.1206,0.1249]	0.1344 [0.1315,0.1375]
HippoV	−0.7125 [−0.8102, −0.4977]	−0.7176 [−0.7201, −7149]	−0.7103 [−0.7127, −0.7079]
AV45∗HippoV	−0.0019 [−0.0029, −0.0012]	−0.1166 [−0.1200, −0.1132]	−0.0016 [−0.0055, 0.0028]
CSF tau∗HippoV	0.0038 [0.0025,0.0059]	−0.0526 [−0.0565, −0.0490]	0.0028 [−0.0016, 0.0071]

This table summarizes standardized regression coefficient estimates, average, and 95% confidence interval, on the simulated data from the mathematical model that had no cognitive reserve component. Consistent, data points were sampled cross-sectionally from one corresponding time point after baseline from each individual trajectory (consistently across trajectories). Random, data points were sampled from a random time point cross-sectionally from each individual trajectory. Random f. residuals, as in random sampling but with the variance associated with time after baseline removed from main effect variables. AV45, florbetapir amyloid-PET summary score; CSF tau, corticospinal fluid total tau; HippoV, hippocampal volume.

### Overestimation of present interaction effects due to data sampling

For the secondary analysis, data were simulated by the mathematical model that did include an interaction component between biomarkers representing cognitive reserve. As in the main analysis, regression models yielded estimates of comparable magnitude for main effects (i.e., biomarkers) on cognitive outcome across the differently sampled simulated data. Specifically, for cross-sectional sampling that was consistent with respect to the time point of the data, the average standardized regression coefficients were 0.2326, 0.2014, and −0.6360 for amyloid-PET, CSF tau, and hippocampal volume, respectively. For cross-sectional sampling that was random with respect to the time point of the data, average standardized regression coefficients were 0.2167, 0.1853, and −0.6107 for amyloid-PET, CSF tau, and hippocampal volume, respectively. For cross-sectional sampling that was random with respect to the time point of the data and with the effect of time partialled out of variables, average standardized regression coefficients were 0.2358, 0.2051, and −0.6053 for amyloid-PET, CSF tau, and hippocampal volume, respectively. However, interaction effect estimates differed between differently sampled simulated data samples. Specifically, for cross-sectional sampling that was consistent with respect to the time point of the data, average standardized regression coefficients were −0.1168 and −0.1315 for interactions of amyloid-PET and CSF tau with hippocampal volume, respectively. In contrast, for cross-sectional sampling that was random with respect to the time point of the data, average standardized regression coefficients were ˗0.2272 and ˗0.1953 for amyloid-PET with hippocampal volume and CSF tau with hippocampal volume, respectively. These interaction effects were such that at higher levels of hippocampal volume the detrimental effect of high amyloid-PET and CSF tau levels on cognitive outcome was attenuated. However, these interaction effects decreased when repeating the random sampling but partialing the effect of time out of variables prior to estimating regression models. The average standardized regression coefficients for this analysis were −0.1222 and −0.1394 for amyloid-PET with hippocampal volume and CSF tau with hippocampal volume and thus comparable to those from the consistent sampling strategy. See Table 3 for an overview.

**Table 3:** Secondary analysis regression estimates

Model term	Consistent	Random	Random f. residuals
AV45	0.2326 [0.1468,0.2576]	0.2167 [0.2146,0.2188]	0.2358 [0.2317,0.2396]
CSF tau	0.2014 [0.0967,0.2799]	0.1853 [0.1831,0.1874]	0.2051 [0.2011,0.2094]
HippoV	−0.6360 [−0.6735, −0.4790]	−0.6107 [−0.6144, −0.6065]	−0.6053 [−0.6094, −0.6015]
AV45∗HippoV	−0.1168 [−0.1442, −0.0115]	−0.2272 [−0.2303, −0.2244]	−0.1222 [−0.1280, −0.1167]
CSF tau∗HippoV	−0.1315 [−0.1667, −0.0033]	−0.1953 [−0.1987, −0.1921]	−0.1394 [−0.1446, 0.1339]

This table summarizes standardized regression coefficient estimates, average, and 95% confidence interval, on the simulated data from the mathematical model that had an added cognitive reserve component. Consistent, data points were sampled cross-sectionally from one corresponding time point after baseline from each individual trajectory (consistently across trajectories). Random, data points were sampled from a random time point cross-sectionally from each individual trajectory. Random f. residuals, as in random sampling but with the variance associated with time after baseline removed from main effect variables. AV45, florbetapir amyloid-PET summary score; CSF tau, corticospinal fluid total tau; HippoV, hippocampal volume.

## Discussion

The results of the present study demonstrate how interaction effects in regression analyses can arise as a statistical artifact due to cross-sectional sampling. The faux interaction effects were demonstrated using simulated data that was generated by a mathematical AD biomarker cascade model, whose parameters were estimated from longitudinal empirical data and whose predictions showed substantial agreement with empirical data points. Furthermore, the faux interaction effects were greatly reduced when partialing out the variance associated with time point of sampling from main effect variables. This finding has important implications for the inferences drawn from interaction effects that could be deemed to indicate cognitive reserve. To our knowledge, this issue has not been extensively discussed prior to this publication.

### Model assessment

Previous studies have employed similar methods, i.e. differential equation models, to model AD. However, some of these studies focused on specific aspects of the disease process whereas most arguably used conceptually as well as mathematically more elaborate models.^11^^,^^12^^,^^13^^,^^14^^,^^15^ In contrast, for the present study, we aimed to demonstrate a statistical artifact through a mathematical model that was as simple as possible in order to be able to argue that it may be present in more complex conceptions of AD as well. The mathematical model’s parameters, i.e. the associations between biomarkers and cognitive reserve as well as their change over time, were estimated from empirical data and are generally in agreement with prior studies. Specifically, the deterioration of all biomarkers over time for patients of AD, i.e. increase of cerebral amyloid and CSF tau, hippocampal atrophy, and cognitive decline, has been demonstrated repeatedly. The agreement of the simulated data points produced by the mathematical model and corresponding empirically available data points was substantial both for biomarkers as well as cognitive outcome, as indicated by standardized regression coefficients ranging between 0.741 and 0.977. As the corresponding $R^{2}$ values ranged between 0.49 and 0.92, we believe the model captures the majority of the temporal evolution of the biomarkers and cognitive outcome considered within the sample and the available time span from 6 to 186 months after baseline.

### Faux interaction effects

**Figure 3:**
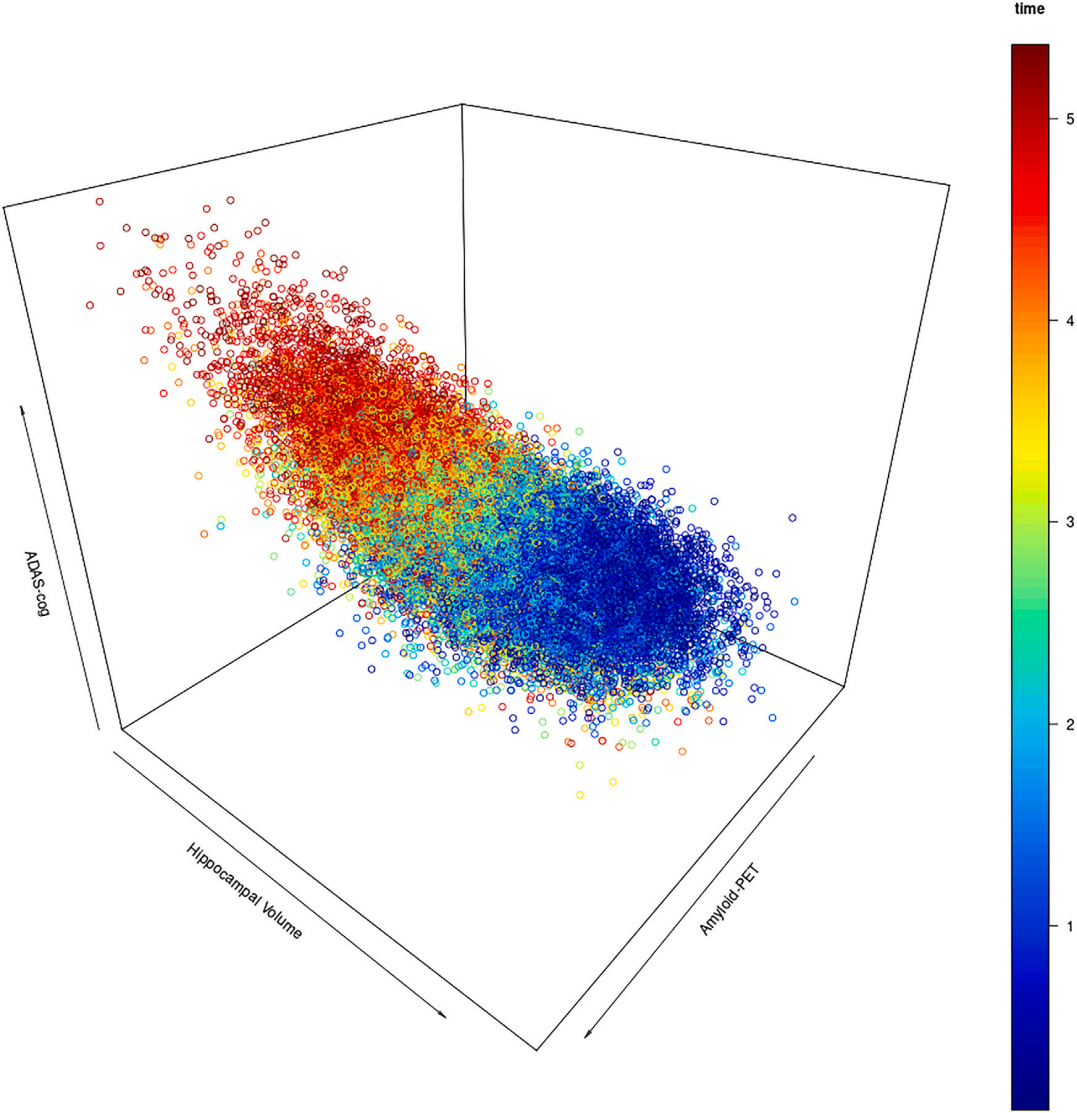
Simulated data points for amyloid-PET and hippocampal volume Scatter plot of simulated data points, with one random time point selected for each initial configuration. Simulation time after baseline in years is color coded. At later time points, cognitive outcome tends to be worse and amyloid-PET and hippocampal volume are jointly altered under the AD biomarker cascade model. Randomly sampling one time point for each simulated subject from this distribtion may lead to the estimation of interaction effects between amyloid-PET and hippocampal volume that are driven by the underlying temporal dynamic rather than cognitive reserve. ADAS-cog, Alzheimer’s disease cognitive assessment scale.

**Figure 4:**
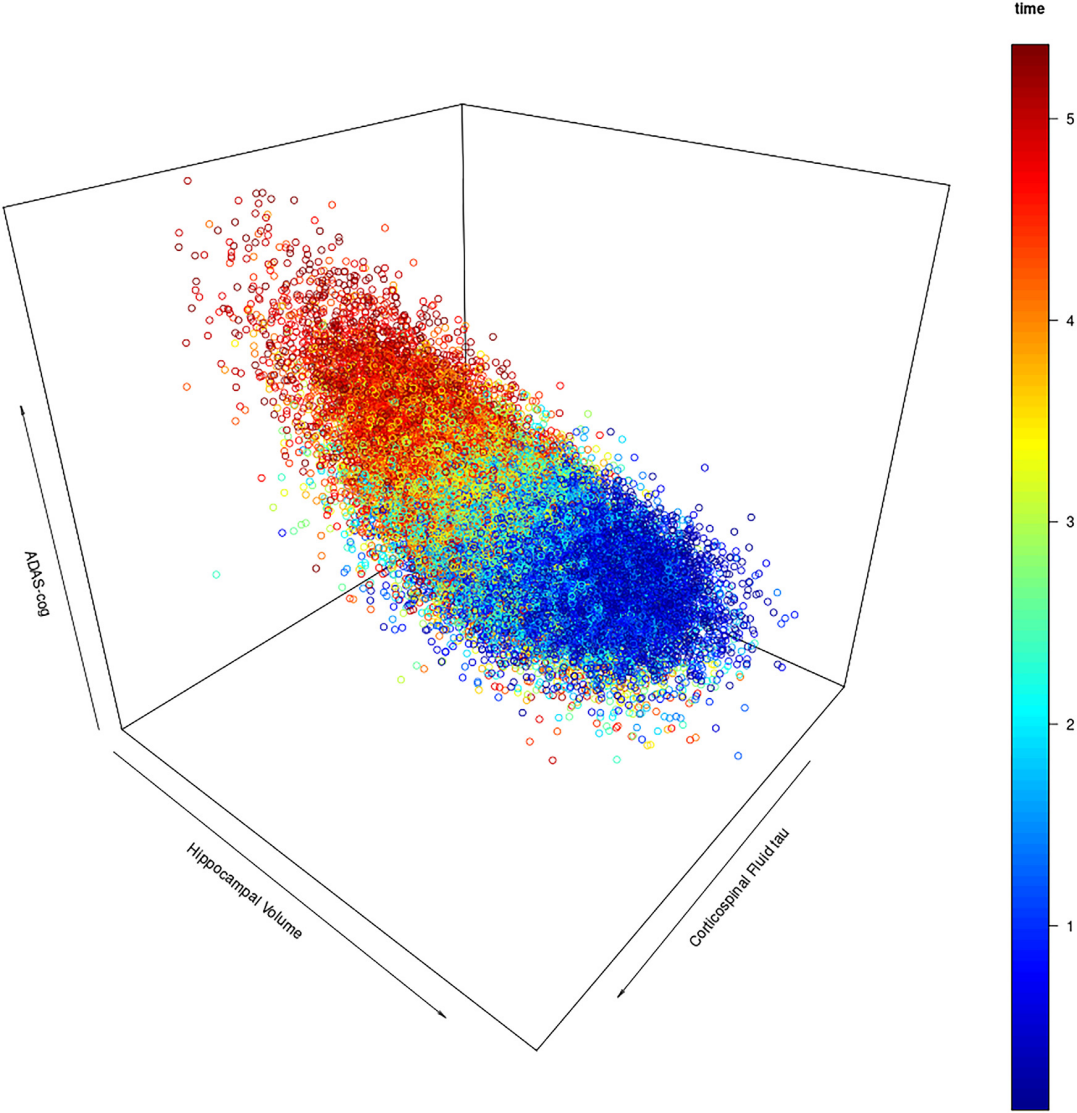
Simulated data points for corticospinalfluid tau and hippocampal volume Scatter plot of simulated data points, with one random time point selected for each initial configuration. Simulation time after baseline in years is color coded. At later time points, cognitive outcome tends to be worse and CSF tau and hippocampal volume are jointly altered under the AD biomarker cascade model. Randomly sampling one time point for each simulated subject from this distribtion may lead to the estimation of interaction effects between amyloid-PET and hippocampal volume that are driven by the underlying temporal dynamic rather than cognitive reserve. ADAS-cog, Alzheimer’s disease cognitive assessment scale.

For the main analysis the mathematical model was explicitly designed not to contain a modulation of biomarkers’ associations with cognitive outcome by other biomarkers. Instead, the mathematical model of the AD biomarker cascade consisted entirely of independent associations between biomarkers and the rate of change of other biomarkers or cognitive outcome. These associations were estimated from empirical data (refer to Equation 2; Table 1). Correspondingly, regression analyses on simulated data, where biomarker levels and cognitive outcome had been estimated at a specific time point after baseline from initial biomarker levels randomly sampled from their empirical multivariate Gaussian distribution, showed independent main effects of biomarkers on cognitive outcome but very small estimates for an interaction effect between biomarkers (see Table 2). However, in naturalistic cross-sectional designs, individually varying levels of biomarkers at the beginning of the disease process are confounded with a measurement time point relative to the beginning of the disease process, which is therefore difficult to estimate and take into account. This circumstance was integrated into the aforementioned analysis by randomly varying the time point after baseline at which the mathematical model simulated biomarker levels and cognitive outcomes. The consequent appearance of interaction effects between biomarkers on cognitive outcome when repeating regression analyses demonstrates that when the measurement time point relative to the disease process is not taken into account, interaction effects between biomarkers may arise purely as a statistical artifact. Indeed, they are greatly diminished when partialing the effect of time after baseline out of variables and repeating this analysis. The mechanism by which these faux interaction effects arise is the following. For the simulated data produced by the mathematical model, the following can be said to be generally true based on the estimated parameters shown in Table 1. First, cognitive outcome will be worse at later points in time after baseline. Second, all biomarkers will also be at worse levels at later time points after baseline. Third, this implies that data points where one biomarker is more altered than the others are at earlier time points relative to baseline. A design where one measures biomarkers and cognitive outcome at a random time point for each individual’s trajectory would consequently yield the following statistical observations for an interaction analysis disregarding the time points of measurements: data points where only one of the main effect variables are altered correspond to better cognitive performance, because they are earlier in time. Data points, where both main effects are altered would correspond to worse cognitive performance than explained by the biomarkers’ individual effects due to being later in time relative to baseline. In other words, time after the beginning of the disease process confounds the interaction term of biomarkers. Please refer to Figures 3 and 4 for visualization.

### Conclusion for the inference on cognitive reserve

Statistical interaction effects have been proposed and used to identify biomarkers of neurophysiological cognitive reserve surrogates that facilitate functional adaption to the presence of pathology.^7^ However, under the premise of the AD biomarker cascade model, statistical interaction effects between biomarkers may arise as an artifact of the data sampling strategy. Specifically, the levels of biomarkers, both of pathology or candidate surrogates of cognitive reserve, and cognitive outcome are a convolute of individually differing initial predispositions (potentially representing brain maintenance and brain reserve) and the amount of time that they have been affecting each other according to the AD biomarker cascade model. Even though associations among biomarkers and cognitive outcome are constant under the biomarker cascade model, data points from later in the disease process will show stronger conjoint alterations of biomarkers and cognitive outcome and may thus drive statistically estimated interaction effects when mixed with data points from earlier in the disease process in cross-sectional designs. For individual data points, time spent under the regime of the biomarker cascade model’s associations among biomarkers and cognitive outcome thus should to be incorporated in some form into statistical analyses. To this end, the literature offers a great deal of staging approaches for AD (refer to the study by Therriault et al.^16^ for a recent review). Even if the disease stage—or time under disease regime—estimates will inevitably be fraught with inaccuracies, assuming independent Gaussian error distribution, they will remain effective to some degree to suppress or mitigate faux interaction effect estimates, see Figure 2. Furthermore, our secondary analysis demonstrates—in principle—that even if the interaction effect investigated represents a convolute of a faux interaction effect due to random cross-sectional sampling and actual underlying cognitive reserve, controlling for the sampling time of each data point could maintain sensitivity to cognitive reserve (see Table 3).

### Limitations of the study

This study has several limitations. Importantly, the mathematical model is kept simple for the sake of argument. In reality, associations among biomarkers may not be constant over time or between individuals. Their effect on cognition may also vary individually due to underlying effects of actual cognitive reserve. Empirical studies also demonstrated an attenuation of the rate of biomarker alteration at higher levels that was not modeled in this study. However, in spite of these simplifications, the agreement of data points simulated based on initial conditions and empirical data is rather good in the present sample. Furthermore, if an attenuation of the rate of alteration of biomarkers and/or lower effects of biomarkers on cognition were present in the model, the basic mechanism for the faux interaction effect of biomarkers of pathology and surrogates of cognitive reserve would remain intact: both would be more altered at later time points along with worse cognition, yielding an interaction effect if time is not taken into account. In addition to limitations pertaining to the model, the sample from which the data have been estimated may not accurately represent the population due to its recruitment schemes. However, this study’s aim is not to make any inferences with regard to the actual disease process of AD nor the prediction of biomarker statuses. Rather, the aim was to demonstrate in a proof-of-concept fashion with a minimal AD mathematical model that misleading statistical interaction effects can arise under the very premise of the core of the AD cascade model.

## Consortia

Data used in preparation of this article were obtained from the Alzheimer’s Disease Neuroimaging Initiative (ADNI) database (adni.loni.usc.edu). As such, the investigators within the ADNI contributed to the design and implementation of ADNI and/or provided data but did not participate in analysis or writing of this report. A complete listing of ADNI investigators can be found at: http://adni.loni.usc.edu/wp-content/uploads/how_to_apply/ADNI_Acknowledgement_List.pdf.

## Resources availability

### Lead contact

Further information and requests for resources and reagents should be directed to and will be fulfilled by the lead contact, Florian U. Fischer (florian.fischer@unimedizin-mainz.de).

### Materials availability

This study did not generate new unique reagents.

### Data and code availability

• This paper analyzes existing, publicly available data. These accession numbers for the datasets are listed in the key resources table.
• All original code is available in this paper’s supplemental information.
• Any additional information required to reanalyze the data reported in this paper is available from the lead contact upon request.

## Acknowledgments

We thank Tatjana Tchumatchenko of the Institute of Physiological Chemistry at the University Medical Center of the Johannes Gutenberg-University Mainz for her aid and insights during the preparation of the manuscript.

Data collection and sharing for this project was funded by the 10.13039/100014041Alzheimer’s Disease Neuroimaging Initiative (ADNI) (10.13039/100000002National Institutes of Health grant U01 AG024904) and DOD ADNI (10.13039/100000005Department of Defense award number W81XWH-12-2-0012). ADNI is funded by the 10.13039/100000049National Institute on Aging, the 10.13039/100000070National Institute of Biomedical Imaging and Bioengineering, and through generous contributions from the following: AbbVie, Alzheimer’s Association; 10.13039/100002565Alzheimer's Drug Discovery Foundation; Araclon Biotech; BioClinica, Inc.; Biogen; Bristol-Myers Squibb Company; CereSpir, Inc.; Cogstate; Eisai Inc.; Elan Pharmaceuticals, Inc.; Eli Lilly and Company; EuroImmun; F. Hoffmann-La Roche Ltd and its affiliated company Genentech, Inc.; Fujirebio; GE Healthcare; IXICO Ltd.; Janssen Alzheimer Immunotherapy Research & Development, LLC.; Johnson & Johnson Pharmaceutical Research & Development LLC.; Lumosity; Lundbeck; Merck & Co., Inc.; Meso Scale Diagnostics, LLC.; NeuroRx Research; Neurotrack Technologies; Novartis Pharmaceuticals Corporation; Pfizer Inc.; Piramal Imaging; Servier; Takeda Pharmaceutical Company; and Transition Therapeutics. The Canadian Institutes of Health Research is providing funds to support ADNI clinical sites in Canada. Private sector contributions are facilitated by the 10.13039/100000009Foundation for the National Institutes of Health (www.fnih.org). The grantee organization is the 10.13039/100009804Northern California Institute for Research and Education, and the study is coordinated by the Alzheimer’s Therapeutic Research Institute at the University of Southern California. ADNI data are disseminated by the Laboratory for Neuro Imaging at the University of Southern California.

## Author contributions

F.F. conceived the paper, conducted the simulation and statistical analyses, and wrote the paper. S.G. and O.T. guided and advised the study conceptually, technically, statistically, and extensively revised the written paper.

## Declaration of interests

The authors declare no competing interests.

## STAR★Methods

### Key resources table

**Table:** 

REAGENT or RESOURCE	SOURCE	IDENTIFIER
**Deposited data**		

Raw and processed data	ADNI	https://adni.loni.usc.edu/

**Software and algorithms**		

R 4.1.2	R Core Team^17^	https://www.R-project.org/
stringr 1.5.0	Wickham^18^	https://CRAN.R-project.org/package=stringr
cAIC 1.0	Säfken et al.^19^	https://CRAN.R-project.org/package=cAIC4
lme4 1.1–31	Bates et al.^20^	https://CRAN.R-project.org/package=lme4
ggplot2 3.4.0	Wickham^21^	https://CRAN.R-project.org/package=ggplot2
ggpubr 0.6.0	Kassambara^22^	https://CRAN.R-project.org/package=ggpubr
mnorm 1.2.2	Potanin^23^	https://CRAN.R-project.org/package=mnorm
plot3D 1.4.1	Soetaert^24^	https://CRAN.R-project.org/package=plot3D
dde 1.0.5	FitzJohn and Hinsley^25^	https://CRAN.R-project.org/package=dde
Simulation implementation	This paper	Data S2

### Experimental model and study participant details

Data used in the preparation of this article were obtained from the Alzheimer’s Disease Neuroimaging Initiative (ADNI) database (adni.loni.usc.edu). The ADNI was launched in 2003 as a public-private partnership, led by Principal Investigator Michael W. Weiner, MD. The primary goal of ADNI has been to test whether serial magnetic resonance imaging (MRI), positron emission tomography (PET), other biological markers, and clinical and neuropsychological assessment can be combined to measure the progression of mild cognitive impairment (MCI) and early Alzheimer’s disease (AD). For up-to-date information, see www.adni-info.org.

#### Subjects

For parameter estimation of the mathematical model, we included subjects of the ADNI cohort that had at least two data points with available Florbetapir (AV45) amyloid-PET summary scores, biospecimen results for corticospinal fluid (CSF) tau, available hippocampal volumetry as well as Alzheimer’s Disease Assessment Scale cognitive outcome scores (ADAS-cog) as per July 2024. For a histogram of the time span of data per subject see supplemental Figure S1. Additionally, subjects were only included if their amyloid-PET summary score was greater or equal to 1.1 at least once. This was to ensure that data points would represent the continuum of the AD-type neurodegenerative trajectories. Demographical data are summarized in below Table. With few exceptions, the ethnicity of the subjects was identified as “white”. Based on the available data, no comment on the socioeconomic status of the subjects could be made.

**Table:** Demographical data

Status	N	Female	Age	Education	Data time span
Preclinical AD	52	32	74.3 ± 5.5	16.4 ± 2.4	90.7 ± 38.6
MCI due to AD	111	50	71.6 ± 6.4	16.1 ± 2.8	73.1 ± 33.5
AD	13	5	76.7 ± 7.0	15.4 ± 3.0	39.7 ± 28.0
Total sample	176	87	72.8 ± 6.4	16.1 ± 2.7	75.8 ± 36.9

This table summarizes the demographical data of the sample used to estimate the mathematical model parameters. Values represent status at the earliest available time point. Age and education in years, data time in months. MCI, mild cognitive impairment. Mean ± SD.

### Method details

#### Neuropsychological assessment

In general, subjects underwent neuropsychological assessment every 12 months over a span of years varying individually. Details regarding the cognitive assessment within the ADNI are given in the publicly available procedures manual: https://adni.loni.usc.edu/wp-content/uploads/2008/07/adni2-procedures-manual.pdf. Within the scope of this study, the cognitive Alzheimer’s Disease Assessment Scale (ADAS-cog), which spans several cognitive domains^26^ was included. The ADAS-cog scale is oriented such that higher scores indicate worse cognitive performance.

#### Corticospinal fluid measurement

CSF biospecimen were stored and analyzed at the Penn ADNI Biomarker Core Laboratory at the University of Pennsylvania, Philadelphia. The multiplex xMAP Luminex platform (Lumnix Corp., Austin, TX) was used to measure CSF concentrations of total tau. For the present study, total tau was included instead of phosphorylated tau, as the latter is included in the former. Total tau thus includes tauopathy specific to AD as well as more general age-related tauopathy that may be relevant for cognitive outcome.^27^ A more detailed description of data collection and processing of the CSF samples can be found in^28^ and at http://adni.loni.usc.edu/methods.

#### Imaging data acquisition and processing

Inversion-recovery spoiled gradient recalled (IR-SPGR) T1-weighted imaging data were acquired on several General Electric 3T scanners using a gradient echo sequence with 11° flip angle and a voxel size of 1.0^2^ × 1.20 mm^3^. AV45 amyloid PET imaging data were acquired on several types of scanners using different acquisition protocols. PET data underwent a standardized preprocessing procedure at the ADNI project to increase data uniformity. Imaging protocols and preprocessing procedures can be accessed at the ADNI website: http://adni.loni.usc.edu/methods/. Using T1-weighted images, total intracranial volume (TIV) as well as hippocampal volume were calculated at ADNI core laboratories using published tissue segmentation methods.^29^^,^^30^^,^^31^ Hippocampal volume was also normalized dividing by the total intracranial volume (TIV). Subjects’ global cortical amyloid load was calculated from AV45 PET images according to ADNI procedures http://adni.loni.usc.edu/methods/pet-analysis/. To this end, cortical amyloid was calculated as the average of the AV45 uptake in the frontal, angular/posterior cingulate, lateral parietal and temporal cortices normalized by dividing by the mean uptake in the cerebellum.^32^

#### Development of a mathematical disease model

Statistical models such as the general linear model allow the inference of interdependencies among biomarkers and their effects on cognition. However, in order to simulate the joint evolution over time of biomarkers, a mathematical model is needed that can predict all involved biomarkers and cognition at any point in time while continuously taking into account their interdependencies. This is achieved in many fields of science using ordinary differential equation (ODE) systems. These are defined by a set of equations that quantify how the rate of change of each variable, in this case biomarkers or cognition, depends on the levels of the other variables at any moment in time. For many such systems, mathematical analysis can produce for these equations a general solution that quantifies explicitly how the level of each variable at any time point depends on the initial conditions. In other words, given an initial state of variables, later states can be exactly calculated given the equation for the general solution of the system. The derivation of the model for this study’s purpose is described more formally as follows: We model the consensus biomarker model of AD mathematically as a linear inhomogeneous differential equation system with constant coefficients. The individual biomarkers as well as the cognitive outcome are represented as system state vector $\vec{x}$, wherein the biomarkers are ordered inversely to their chronological order, i.e., each biomarker comes before the set of biomarkers that precede it in the cascade model. Specifically, the first entry is the cognitive outcome followed by hippocampal volume, CSF tau and amyloid-PET. The associations among the biomarkers are represented by the triangular matrix *A*, and the association with time is represented by $\vec{c}$. The matrix *A* is triangular due to the constraint on hypothetical causality implied by the chronological order of the biomarker cascade model, i.e. a biomarker can only conceivably depend on the biomarkers that precedes it in the cascade model. Hence also the ordering of the state vector $\vec{x}$. The model is thus represented by a differential equation system of the form.

(Equation 1) $$\frac{d\vec{x}}{dt}=A\vec{x}+\vec{c}$$

#### Determining the model parameters

We determined the association of the *i* individual biomarkers with each other and with time using linear mixed effects models (see section quantification and statistical analysis below for details). The estimated regression coefficients were inserted in equations representing the association of each biomarker $x_{i}$ with time *t*, the biomarkers preceding it in the biomarker cascade $x_{j}$ as well as the modulation of the association with time by the respective biomarkers $x_{j}t$, where j>i. These equations take the form

(Equation 2) $$x_{i}=\alpha_{i}t+\sum_{j>i}^{n}\beta_{i,j}x_{j}+\sum_{j>i}^{n}\gamma_{i,j}x_{j}t$$

Their derivatives are

(Equation 3) $$\frac{dx_{i}}{dt}=\alpha_{i}+\sum_{j>i}^{n}\gamma_{i,j}x_{j}$$

We can thus set the model parameters by inserting them into Equation 1, setting the matrix *A*’s elements $a_{i,j}=\gamma_{i,j}$ and the inhomogeneous component $c_{i}=\alpha_{i}$.

#### Analytical solution

The solution to a linear inhomogeneous differential equation system has the form

(Equation 4) $$\vec{x}\left(t\right)=e^{tA}{\vec{x}}_{0}+e^{tA}\int_{0}^{t}e^{-sA}\vec{c}\left(s\right)ds$$

We can calculate the matrix exponential exactly using the Taylor series, which aborts after a finite number of terms as *A* is a triangular and thus nilpotent matrix. Additionally, as $\vec{c}\left(s\right)$ is time independent in our model, it can be simplified to $\vec{c}$. Substituting the matrix exponentials with their Taylor series expansion and calculating the integral then yields the solution to the model

(Equation 5) $$\vec{x}\left(t\right)=\sum_{k=0}^{n-1}\frac{t^{k}}{k!}A^{k}\vec{x_{0}}+\left(\sum_{k=0}^{n-1}\frac{t^{k}}{k!}A^{k}\right)\left(\sum_{k=0}^{n-1}\frac{{\left(-1\right)}^{k}}{k!\left(k+1\right)}t^{k+1}A^{k}\vec{c}\right)$$

#### Addition of a cognitive reserve component

For the secondary analysis, where we aimed to demonstrate that an interaction effect refferring to cognitive reserve could in principle be detected in cross sectional data (see following paragraph), we expanded the mathematical model for a term to incorporate an interaction effect between amyloid-PET and hippocampal volume as well as CSF tau and hippocampal volume, such that at higher levels of hippocampal volume, cognitive outcome would deteriorate less due to higher amyloid-PET or CSF tau over time. To this end, Equation 1 was modified to

(Equation 6) $$\frac{d\vec{x}}{dt}=A\vec{x}+\vec{c}+\left[\begin{array}{l} -.1\left(x_{2}x_{3}+x_{2}x_{4}\right)\\ 0\\ 0\\ 0 \end{array}\right]$$

where $x_{2}$ represents hippocampal volume, $x_{3}$ CSF tau and $x_{4}$ amyloid-PET. This system was integrated numerically using dopri5.^33^

### Quantification and statistical analysis

Empirical data were z-standardized throughout. In order to define the mathematical model parameters, we estimated for each biomarker and the cognitive outcome score as dependent variable a family of nested linear mixed effects models consisting of every possible combination of the biomarkers preceding the respective biomarker/the cognitive outcome score in the biomarker cascade as well as their interaction terms with time as predictor, as well as the model without other biomarkers. Time (in months after baseline), the covariates age and education (in years) as well as as a random intercept for each subject were included in all models. Using conditional Akaike Information Criterion,^34^^,^^35^ the model best describing the data was then selected and its estimates used to set the model parameters as described above. If a preceding biomarker’s interaction term was not included in the best fitting model, its corresponding entry in *A* was set to zero. In order to assess the correspondence of the simulated data produced by the mathematical model with the available empirical data points, we used the biomarker and cognitive outcome data of each subject at baseline (if available) to calculate the model predictions of cognitive outcome and biomarker statuses at subsequent time points that were also available in the real data. These simulated data points were then compared to the corresponding empirical data via simple linear regression models, with the mathematical model predictions as the predictor and the empirical data as dependent variable. The aim of the main analysis was to demonstrate the emergence of interaction effects due to cross sectional sampling of data simulated under the premise of the AD biomarker cascade model (see above). To this end, we first simulated individual biomarker and cognitive status trajectories over time in the following way. Using the earliest available time points with complete biomarker data for each subject in the empirical data, we calculated the parameters of the multivariate Gaussian distribution of amyloid-PET, CSF tau, hippocampal volume and cognitive outcome ADAS-cog. Based on this distribution, we randomly generated 50,000 data points which yielded the set of all initial configurations. Subsequently, for each of these initial configurations, biomarker and cognitive outcome status were calculated for the time points that were measured in the empirical data - 6 to 186 months after baseline - using the mathematical model described above. Second, we estimated regression models with cognitive outcome as dependent variable, the biomarkers as main effects and the interaction terms of amyloid-PET with hippocampal volume and CSF tau with hippocampal volume. The regression models were estimated on two differently sampled subsets of the simulated individual trajectories described in the previous paragraph, one representing a cross-sectional analysis informed of the time-correspondence of data points, where a regression model was estimated for each calculated time point after baseline on the simulated data points corresponding to the respective time point (consistent sampling). The other represented a ’naturalistic’ cross-sectional analysis uninformed of the time-correspondence, where one simulated data point was sampled for each individual trajectory at a random time point (random sampling). The random sampling and regression estimation was repeated 1000 times for this second analysis and estimated regression coefficients were averaged. This analysis was then repeated with the effect of time partialled out of each variable by using each variable’s residuals from a simple regression with the respective variable as dependent and time after baseline as independent variable (random sampling from residuals). As real world estimates of time relative to the disease process will likely be subject to inaccuracies, we repeated this last step with Gaussian noise added to the time variable. The standard deviation parameter of the noise was scaled from 5% to 100%, increasing in steps of 5%, of the maximum time interval of the simulation, i.e., 186 months. Finally, for the secondary analysis we repeated the main analysis as described in the previous two paragraphs using the exact same parameters but with a mathematical model that had an added term to model an interaction of hippocampal volume with amyloid-PET and with CSF tau. This was done in order to investigate whether controlling for the faux interaction effect would in principle leave actual underlying interactions referring to cognitive reserve detectable by conventional statistics. For each set of regression estimates, i.e. consistent sampling, random sampling and random sampling from residuals, we reported the 95% confidence interval and the average of regression coefficient estimates (see Tables 2 and 3).

#### Software packages

For the implementation of the mathematical model, simulation and statistical data analysis as described above, we used R version 4.1.2^17^ with the following packages: stringr version 1.5.0,^18^ cAIC4 version 1.0,^19^ lme4 version 1.1–31,^20^ ggplot2 version 3.4.0,^21^ ggpubr version 0.6.0,^22^ mnorm version 1.2.2,^23^ plot3D version 1.4.1^24^ and dde version 1.0.5.^25^

## Supplemental information

Document S1. Figure S1

Data S2. This file contains the R code implementing the complete analysis, related to the STAR Methods section
