# Supplementary material for: Mathematical model of the Alzheimer’s disease biomarker cascade demonstrates statistical pitfall in identifying surrogates of cognitive reserve
Source: iScience. 2024 Oct 18;27(11):111188. doi: 10.1016/j.isci.2024.111188 (PMC11607535; doi:10.1016/j.isci.2024.111188)
Supplement: Document S1. Figure S1 [file mmc1.pdf]

iScience, Volume *27*

Supplemental information

Mathematical model of the Alzheimer's disease biomarker cascade demonstrates statistical pitfall in identifying surrogates of cognitive reserve

Florian U. Fischer, Susanne Gerber, Oliver Tüscher, and for the Alzheimer's Disease Neuroimaging Initiative

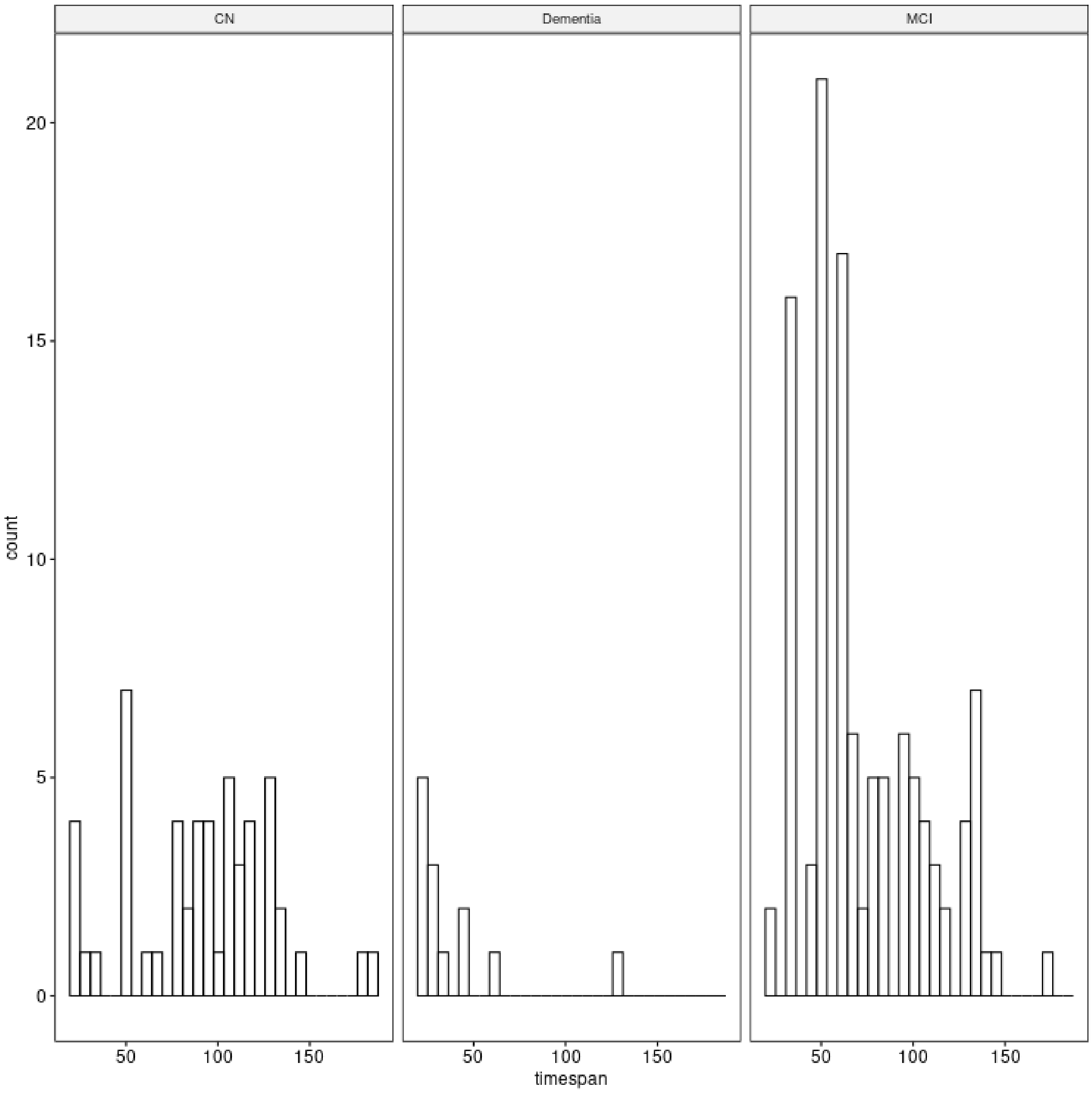

Figure S1. Histogram of timespan from baseline to the last available empirical data point for each individual by clinical status at baseline. CN, cognitively normal. Dementia, manifest Alzheimer's disease. MCI, mild cognitive impairment. Timespan in months.
